# Th2 Cell-Intrinsic Hypo-Responsiveness Determines Susceptibility to Helminth Infection

**DOI:** 10.1371/journal.ppat.1003215

**Published:** 2013-03-14

**Authors:** Nienke van der Werf, Stephen A. Redpath, Miyuki Azuma, Hideo Yagita, Matthew D. Taylor

**Affiliations:** 1 Department of Experimental Immunology, Academic Medical Center, University of Amsterdam, Amsterdam, The Netherlands; 2 Department of Microbiology and Immunology, University of British Columbia, Vancouver, British Columbia, Canada; 3 Department of Molecular Immunology, Tokyo Medical and Dental University, Tokyo, Japan; 4 Department of Immunology, Juntendo University School of Medicine, Tokyo, Japan; 5 Institute of Immunology and Infection Research, School of Biological Sciences, University of Edinburgh, Edinburgh, United Kingdom; 6 Centre for Immunity, Infection and Evolution, School of Biological Sciences, University of Edinburgh, Edinburgh, United Kingdom; New York University, United States of America

## Abstract

The suppression of protective Type 2 immunity is a principal factor driving the chronicity of helminth infections, and has been attributed to a range of Th2 cell-extrinsic immune-regulators. However, the intrinsic fate of parasite-specific Th2 cells within a chronic immune down-regulatory environment, and the resultant impact such fate changes may have on host resistance is unknown. We used IL-4gfp reporter mice to demonstrate that during chronic helminth infection with the filarial nematode *Litomosoides sigmodontis*, CD4^+^ Th2 cells are conditioned towards an intrinsically hypo-responsive phenotype, characterised by a loss of functional ability to proliferate and produce the cytokines IL-4, IL-5 and IL-2. Th2 cell hypo-responsiveness was a key element determining susceptibility to *L. sigmodontis* infection, and could be reversed in vivo by blockade of PD-1 resulting in long-term recovery of Th2 cell functional quality and enhanced resistance. Contrasting with T cell dysfunction in Type 1 settings, the control of Th2 cell hypo-responsiveness by PD-1 was mediated through PD-L2, and not PD-L1. Thus, intrinsic changes in Th2 cell quality leading to a functionally hypo-responsive phenotype play a key role in determining susceptibility to filarial infection, and the therapeutic manipulation of Th2 cell-intrinsic quality provides a potential avenue for promoting resistance to helminths.

## Author Summary

Helminth parasites mount chronic infections in over 1 billion people worldwide, of which filarial nematode infections account for 120 million. A major barrier to the development of protective Th2 immunity lies in the dominant down-regulatory immune responses invoked during infection. Although this immune suppression is linked with a range of Th2 cell-extrinsic immune regulators, the fate of CD4^+^ Th2 cells during chronic infection, and the role of Th2 cell-intrinsic regulation in defining protective immunity to infection is largely unknown. In this study, we use a murine model of filarial nematode infection to show that as infection progresses the Th2 effector cells responsible for killing helminths become functionally hypo-responsive, developing a phenotype similar to adaptive tolerance or exhaustion, and their ability to clear infection becomes impaired. We further demonstrate that we can therapeutically manipulate the intrinsic functional quality of hypo-responsive Th2 cells via the PD-1/PD-L2 co-inhibitory pathway to reawaken them and enhance resistance to infection. Thus, our data provide the first demonstration that Th2 cell-intrinsic hypo-responsiveness plays a key role in determining susceptibility to helminth infection.

## Introduction

Protective immunity to helminth parasites takes decades to acquire, if it develops at all, with over 1 billion people harbouring chronic infections [1]. Protection is mediated by the Th2 arm of immunity [2], which is also responsible for causing allergic diseases such as asthma, atopic dermatitis, and allergic rhinitis, and types of fibrosis. A major reason for the failure in anti-helminth Th2 immunity is that the parasites immunosuppress their host, exemplified by host PBMC losing the ability to proliferate and produce Th2 cytokines, such as IL-4 and IL-5, in response to parasite antigen [3], [4], [5]. Interestingly, this Th2 down-modulation has parallels with the modified Th2 response originally described in association with tolerance to allergens, and characterised by a switch from an inflammatory IgE response to an anti-inflammatory IgG4 and IL-10 response [6], [7]. Thus, the regulatory pathways invoked by helminths can cross-regulate and protect against allergic diseases in humans and animal models [8], [9]. As such, defining the mechanisms of immune down-regulation during helminth infections is of importance for the development of therapeutic strategies or vaccines to induce long-term protective anti-helminth immunity, and novel approaches for the treatment of allergies and fibrosis.

Following the observations that neutralisation of IL-10 or TGF-β can restore the immune-responsiveness of PBMC from helminth-infected individuals [10], [11], studies have focussed on determining the extrinsic regulators that control Th2 cell function. From these, a variety of cell types have been shown to inhibit immunity to helminths and allergens [12], including Foxp3^+^ regulatory T cells (Tregs) [13], [14], alternatively activated macrophages (AAM) [15], [16], DC [17], [18], and B cells [19], [20]. However, the intrinsic fate of parasite-specific CD4^+^ Th2 cells within a chronic down-regulatory environment is largely unknown, even though the idea that helminth-elicited T cells become anergised during infection was postulated 20 years ago [21]. It is known that CD8^+^ T cells develop a functionally hypo-responsive phenotype in chronic Th1 infections, termed exhaustion [22], and human helminth studies provide some evidence for the development of a form of Th2 cell-intrinsic dysfunction. PBMC from filariasis patients display a gene expression profile characteristic of anergic T cells [3], and T cells from individuals with chronic nematode infections show defects in TCR signalling [23]. Recently, a murine study on the down-modulation of pathogenic Th2 responses during *Schistosoma mansoni* infection provided the first formal demonstration that CD4^+^ Th2 effector cells can develop an intrinsically hypo-responsive phenotype [24]. Thus, there is a question of whether individuals fail to acquire protective immunity to helminths because their Th2 cells become intrinsically dysfunctional.

We previously used a murine model of filariasis, *Litomosoides sigmodontis* infection of permissive BALB/c mice, to define the immune regulatory mechanisms that prevent helminth killing. We demonstrated that purified CD4^+^ T cells lose the ability to proliferate and produce Th2 cytokines to parasite antigen as infection progresses [25]. This loss of function within the CD4^+^ T cell compartment was independent of Foxp3^+^ Tregs and IL-10 indicating that, alongside extrinsic regulation by Foxp3^+^ Tregs [24], [25], [26], susceptibility to filarial infection is associated with an intrinsic functional change within the CD4^+^ Th2 cells. Thus, in this study we employed IL-4gfp 4get reporter mice [27] to track and determine the fate of Th2 cells during *L. sigmodontis* infection. We found that, whilst increasing in number, the IL-4gfp^+^CD4^+^ Th2 cells became conditioned towards a functionally hypo-responsive phenotype as infection progressed denoted by a progressive loss in their intrinsic ability to produce the cytokines IL-4, IL-5 and IL-2. The onset of hypo-responsiveness was accompanied by increased expression of PD-1 by IL-4gfp^+^ Th2 cells, and in vivo PD-1 blockade led to increased resistance to infection and a long-term increase in Th2 cell functional quality. In contrast to viral and protozoan infections [28], [29], [30], the control of T cell quality by PD-1 was driven through its interactions with PD-L2, and not PD-L1. Thus, intrinsic changes in Th2 cell functional quality play an important role in defining resistance and susceptibility to filarial nematodes, and it is possible to enhance resistance to infection by therapeutically manipulating Th2 cell quality.

## Results

### Th2 cells are conditioned towards a functionally hypo-responsive phenotype during chronic infection with *Litomosoides sigmodontis*

Susceptibility to *L. sigmodontis* infection is associated with a loss of responsiveness by CD4^+^ T cells at the infection site, the pleural cavity (PC), such that as infection progresses purified PC CD4^+^ T cells show reduced *L. sigmodontis* antigen (LsAg)-specific proliferative and cytokine responses in vitro [25], [26]. This down-modulation within the CD4^+^ T cell population was independent of extrinsic regulation by CD4^+^CD25^+^Foxp3^+^ Tregs or IL-10, suggesting that it represented either a contraction in the number of Th2 cells, or a qualitative change in the intrinsic function of the responding Th2 cells.

Identifying parasite-specific T cells during helminth infection is challenging due to the polyclonal nature of the response and a lack of knowledge of the specific antigens recognised. Thus, to determine whether changes in Th2 cell quantity or intrinsic functional quality explain the observed CD4^+^ T cell down-modulation during *L. sigmodontis* infection we employed BALB/c IL-4gfp 4get reporter mice [27]. IL-4gfp^+^ T cells elicited during acute infection with the nematode *Nippostrongylus brasiliensis* are parasite specific [31], and so IL-4gfp expression by CD4^+^ T cells was used as a tool for tracking *L. sigmodontis*-specific Th2 cells, with a caveat that IL-4gfp is a surrogate marker and a small proportion of IL-4gfp^+^ T cells may not be *L. sigmodontis* specific. Importantly, once committed to the Th2 lineage, T cells store IL-4 mRNA and the production of IL-4 protein is controlled post-transcriptionally [32]. IL-4gfp 4get mice report the presence of IL-4 mRNA independently of IL-4 protein [32], [33], meaning that the number of committed IL-4 mRNA^+^ Th2 cells can be quantified by GFP expression, whilst further assays can be used to independently assess their functional quality.

**Figure 1:**
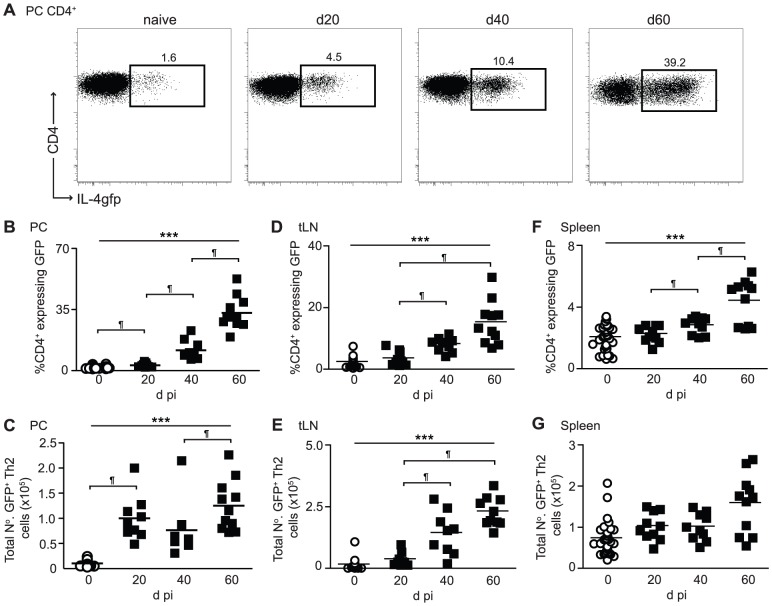
The proportion and total number of IL-4gfp^+^ Th2 cells increase as infection progresses. PC, tLN, and splenic CD4^+^ T cells from naïve (open symbols) and *L. sigmodontis* infected (closed symbols) BALB/c IL-4gfp mice were analyzed at d20, d40 and d60 pi for expression of GFP. (A) Representative flow plots showing expression of CD4 versus GFP by PC CD4^+^ T cells. (B–G) Percentage of CD4^+^ T cells expressing GFP (B, D, F) and total numbers of IL-4gfp^+^ Th2 cells (C, E, G) within the PC (B & C), tLN (D & E) and spleen (F & G). Symbols represent individual mice and lines represent means. Panels show pooled data from two independent experiments, with 4–6 mice per group. *** Significant increase over time (p<0.001, ANOVA using combined data from two experiments), ¶ significant pair-wise comparison (p<0.05, Tukey's HSD).

BALB/c IL-4gfp mice were infected s.c. with *L. sigmodontis* larvae. From the skin the larvae migrate via the lymphatics to the PC by d 4 post-infection (pi) where they undergo a series of moults reaching the adult stage around d 25 pi, and become sexually mature with the females releasing transmission stage microfilaria (Mf) approximately 55 d pi. A patent infection is defined as having mature adult parasites within the PC, and Mf circulating within the blood stream [34]. At the infection site there was a gradual increase in the proportion of IL-4gfp^+^ Th2 cells as infection progressed, culminating in 35% of CD4^+^ T cells expressing GFP by day 60 (Figure 1A and B). This translated to a significant elevation of total numbers of IL-4gfp^+^ Th2 cells by d 20 pi, which was maintained until d 60 pi (Fig. 1C). Increases in the proportions of IL-4gfp^+^ Th2 cells were also seen in the thoracic LN (tLN) and spleen (Figure 1D and F), albeit to a lesser extent, and only resulted in significantly increased total numbers of IL-4gfp^+^ Th2 cell in the tLN (Figure 1E and G). Thus, the down-modulation of CD4^+^ T cell responsiveness within the PC was not caused by a loss of Th2 cells.

**Figure 2:**
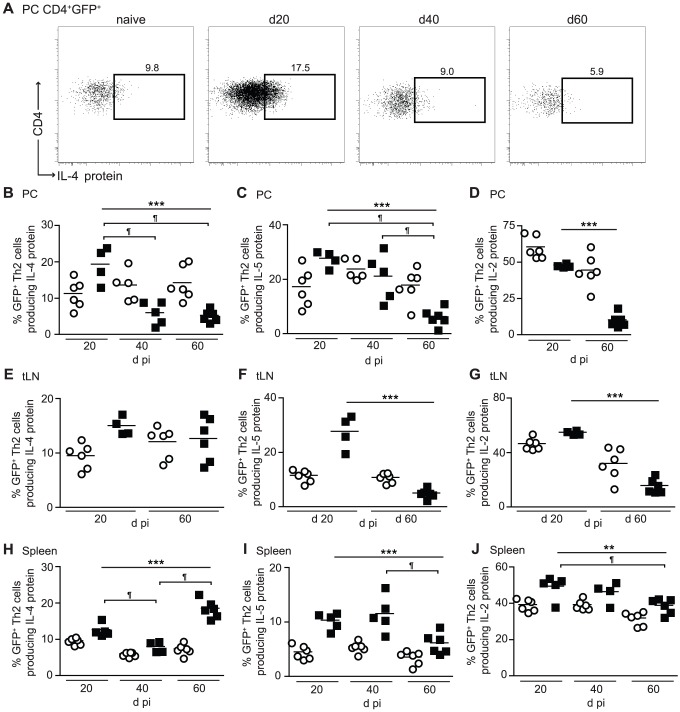
CD4^+^ Th2 cells are conditioned towards a functionally hypo-responsive phenotype during *L. sigmodontis* infection. PC, tLN, and splenic CD4^+^ T cells from naïve (open symbols) and *L. sigmodontis* infected (closed symbols) BALB/c IL-4gfp mice were analyzed at d20, d40 and d60 pi for expression of GFP, IL-4, IL-5 and IL-2. (A) Representative flow plots showing expression of IL-4 protein by PC IL-4gfp^+^CD4^+^ T cells. (B–J) Percentage of PC (B–D), tLN (E–G), and splenic (H–J) IL-4gfp^+^CD4^+^ Th2 cells producing IL-4 (B, E, H), IL-5 (C, F, I), and IL-2 (D, G, J) protein upon stimulation with PMA and ionomycin. Symbols represent individual animals and lines represent means. Panels show one representative experiment of two with 4–6 mice per group. ***p<0.001, ** p<0.05, significant change over time (ANOVA performed using combined data from infected mice from two experiments), ¶ significant pair-wise comparison (p<0.05, Tukey's HSD).

To determine whether Th2 cell functional quality declined during infection, intra-cellular staining was used to define the proportion of IL-4gfp^+^ Th2 cells actively producing IL-4 protein (Figure 2A), as well as IL-5 and IL-2 (Figure S1), in response to PMA and ionomycin stimulation. Contrasting with the increase in numbers of IL-4gfp^+^ Th2 cells within the PC, there was a 69% reduction in the proportion of IL-4gfp^+^ Th2 cells making IL-4 protein between d 20 and d 40, which was still apparent at d 60 (Figure 2B). The proportion of IL-4gfp^+^ Th2 cells producing IL-5 protein also declined by 70%, although with delayed kinetics as the reduction did not occur until d 60, indicating a staggered loss of cytokine production (Figure 2C). Similarly, there was a 79% decrease in the proportion making IL-2 between d 20 and d 60 (Figure 2D). Distal to the PC, the proportion of IL-4gfp^+^ Th2 cells capable of producing IL-4 protein within the tLN remained unaffected (Figure 2E), and despite a transient decrease at d 40 in the spleen the proportion of IL-4^+^ IL-4gfp^+^ Th2 cells at d 60 pi was equivalent to d 20 (Figure 2H). In contrast, the production of IL-5 and IL-2 proteins by IL-4gfp^+^ Th2 cells was impaired at d 60 in both the tLN and spleen (Figure 2 F, G, I and J).

Thus, the decline in CD4^+^ T cell responsiveness observed during chronic *L. sigmodontis* infection represents a step-wise intrinsic loss of functional ability of IL-4gfp^+^ Th2 cells to produce cytokines, rather than a decrease in the total number or proportion of Th2 cells. This hypo-responsive Th2 cell phenotype is most prominent at the infection site, but to a lesser extent radiates out to the draining LN and spleen.

### Hypo-responsive IL-4gfp^+^ Th2 cells express PD-1

**Figure 3:**
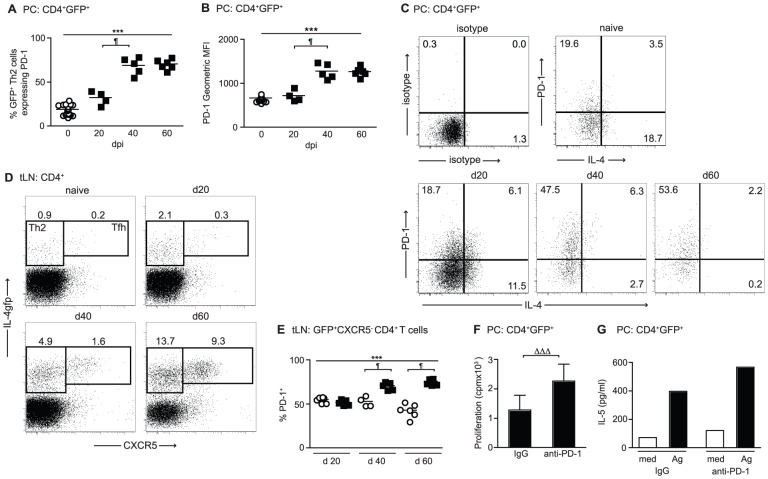
Th2 cell hypo-responsiveness is associated with PD-1. (A–D) PC and tLN CD4^+^ T cells from naïve (open symbols) and *L. sigmodontis* infected (closed symbols) BALB/c IL-4gfp mice were analyzed at d20, d40 and d60 pi for expression of GFP, PD-1, CXCR5, and IL-4. (A–B) Percentage (A) and fluorescence intensity (B) of PD-1 expression by PC IL-4gfp^+^CD4^+^ Th2 cells. (C) Representative plots showing expression of PD-1 versus intra-cellular IL-4 by PC IL-4gfp^+^ Th2 cells. (D) Representative staining for GFP and CXCR5 on tLN CD4^+^ T cells. (E) Percentage of tLN CD4^+^GFP^+^CXCR5^−^ Th2 cells expressing PD-1. Symbols represent individual mice, and lines represent means. Panels show one representative experiment out of two. *** Significant increase over time (p<0.001, ANOVA performed using combined data from two experiments), ¶ significant pair-wise comparison, (p<0.05, Tukey's HSD). (F & G) In vitro proliferation minus medium controls (F) and IL-5 production (G) by PC IL-4gfp^+^ Th2 cells purified from *L. sigmodontis* infected mice 60 d pi and restimulated with LsAg in presence of anti-PD-1 mAb or control rat IgG. IL-4gfp^+^ Th2 cells were pooled from 10–15 mice, and panels show one representative experiment of two. Bars represent means. Due to the necessity for pooling samples, error bars show SD of triplicate cultures for proliferation (F), and it was not possible to calculate error bars or statistics for IL-5 (G). ΔΔΔ p<0.025, (ANOVA performed using combined data from two experiments).

Co-inhibition through the PD-1 pathway leads to the functional exhaustion of CD8^+^ T cells during chronic immune challenge [22], [28], and is involved in the inhibition of Th2 responses during helminth infections [35], [36], [37], [38]. To investigate whether *L. sigmodontis*-induced Th2 cell hypo-responsiveness was associated with PD-1 co-inhibition, the expression of PD-1 by IL-4gfp^+^ Th2 cells was assessed. At d 20 pi, when the IL-4gfp^+^ Th2 cells were still functionally active, there was no change in the proportion of PC IL-4gfp^+^ Th2 cells expressing PD-1 (Figure 3A). However, concomitant with the onset of hypo-responsiveness, there was a two-fold increase in the proportion of IL-4gfp^+^ Th2 cells expressing PD-1 at d 40 pi. PD-1 expression remained elevated until d 60 (Figure 3A), and the mean fluorescence intensity of PD-1 expression on IL-4gfp^+^ Th2 cells increased with similar kinetics (Figure 3B). When PD-1 expression by IL-4gfp^+^ Th2 cells was compared to production of IL-4 protein, the majority of IL-4 producing Th2 cells at d 20 were PD-1 negative, and the enhanced PD-1 expression at d 40 and 60 associated with the loss of IL-4 protein (Figure 3C).

T follicular helper (Tfh) cells are IL-4gfp^+^ and express PD-1 during helminth infection [39], [40], [41], and as expected the increases in tLN IL-4gfp^+^ T cells observed during *L. sigmodontis* infection represented an expansion of both IL-4gfp^+^CXCR5^−^ Th2 cells and IL-4gfp^high^CXCR5^+^ Tfh cells (Figure 3D). IL-4gfp^high^CXCR5^+^ Tfh cells from naïve mice constitutively expressed high levels of PD-1 and there was no change upon infection (data not shown). In contrast, infection with *L. sigmodontis* significantly increased the percentage of IL-4gfp^+^CXCR5^−^ Th2 cells expressing PD-1 from d 40 onwards (Figure 3E).

To test the hypothesis that blocking PD-1 signalling on the hypo-responsive Th2 cells in vitro could re-activate their functional responsiveness, IL-4gfp^+^ Th2 cells were purified from the PC 60 d post-*L. sigmodontis* infection and restimulated with LsAg in the presence of an anti-PD-1 blocking mAb [42] using irradiated naïve splenocytes as APC. Due to the low number of IL-4gfp^+^ T cells present within the PC it was necessary to pool cells from 10–15 mice to obtain sufficient cell numbers. Significantly increased LsAg-specific proliferation and elevated IL-5 production was seen upon addition of the anti-PD-1 mAb (Figure 3F and G), indicating that PD-1 blockade can restore the function of committed Th2 cells. Thus, Th2 cell hypo-responsiveness is associated with increased expression of PD-1 by IL-4gfp^+^ Th2 cells in both the PC and tLN, and blocking PD-1 in vitro increases the antigen-specific capacity of PC IL-4gfp^+^ Th2 cells to proliferate and produce IL-5.

### PD-1 blockade during established infection enhances host resistance to *L. sigmodontis*

**Figure 4:**
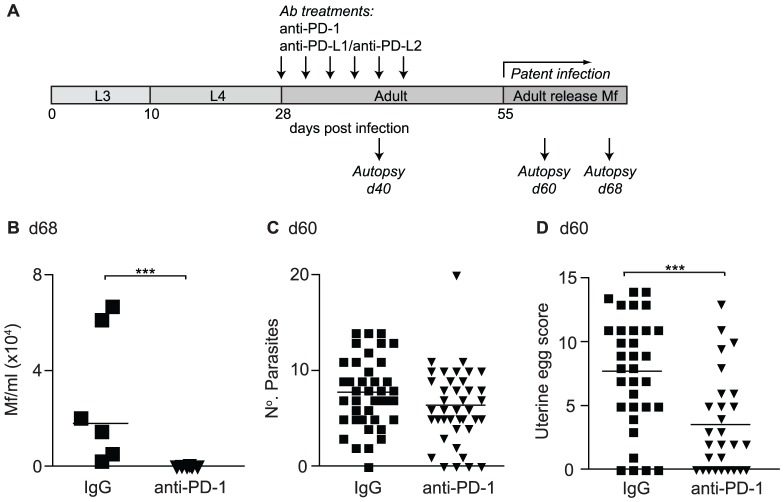
In vivo PD-1 blockade increases resistance to *L. sigmodontis*. (A) Timeline of *L. sigmodontis* infection showing approximate timings of the molts from larval (L3/L4) to adult stages and the development of patency in relation to in vivo antibody treatments and autopsies. (B–D) *L. sigmodontis*-infected BALB/c IL-4gfp reporter mice were treated with a blocking anti-PD-1 mAb (triangles) or rat IgG (squares) from d28–d43 and their adult parasite burdens assessed at d 60 pi, and their blood Mf levels at d 68 pi. (B) Mf counts per ml of peripheral blood. (C) Number of adult parasites within the PC. (D) Number of live eggs within the uteri of individual female parasites recovered from IgG and anti-PD-1 treated hosts. Panels show one representative experiment of two (B) or pooled data from four independent experiments (C & D). Symbols represent individual mice (B & C) or female parasites (D), and lines represent means (B & C) or medians (D). *** p<0.001 (ANOVA performed on combined data from two (B) or four (C & D) independent experiments).

To directly test whether co-inhibition through the PD-1 pathway inhibits protective immunity, *L. sigmodontis* infection was allowed to establish within susceptible BALB/c mice and PD-1 activity blocked from d 28–43 pi using a neutralising anti-PD-1 mAb (Figure 4A). PD-1 blockade impaired the ability of *L. sigmodontis* to develop a fully patent infection as the incidence and levels of blood Mf were significantly reduced in anti-PD-1 treated mice compared to control mice at d 68 pi (Figure 4B and Table 1). As there was no effect of treatment on the number of adult parasites recovered at d 60 pi (Figure 4C), we scored the uterine egg and Mf contents of female parasites to determine whether PD-1 blockade reduced blood Mf by inhibiting fecundity. Changes in helminth fecundity are often a sensitive and quantitative measure of the efficacy of host immunity, even when it is insufficient to kill adult parasites [43]. There was a significant reduction in the number of healthy uterine eggs within female parasites from PD-1 treated mice at d 60 pi (Figure 4D). In addition, the number of female parasites with Mf within their uteri was reduced three-fold following PD-1 treatment (Table 1), although those with uterine Mf tended to have similar levels as female parasites from the IgG controls (Figure S2A). Thus, in vivo PD-1 blockade promotes host resistance to established *L. sigmodontis* infection resulting in impaired fitness and fecundity in a proportion of the female parasites, and reduced levels and incidence of circulating transmission stage Mf within the host's blood.

**Table 1:** In vivo PD-1 blockade results in a reduced incidence of hosts with blood MF and of parasites with uterine Mf.

	Incidence of blood Mf 68 d pi		Incidence of uterine Mf 60 d pi	
	IgG	Anti-PD-1	IgG	Anti-PD-1
Exp 1	4/6	0/6	10/46	3/35
Exp 2	6/6	2/5	7/12	0/18
Exp 3			13/32	5/28
Total	10/12 (83%)	2/11 (18%) *	30/90 (33%)	8/82 (10%)*

* Significant difference in incidence compared to IgG controls, p<0.001 (GLM using combined data from two or three experiments).

### PD-1 blockade results in the long-term re-activation of Th2 cells at the infection site

**Figure 5:**
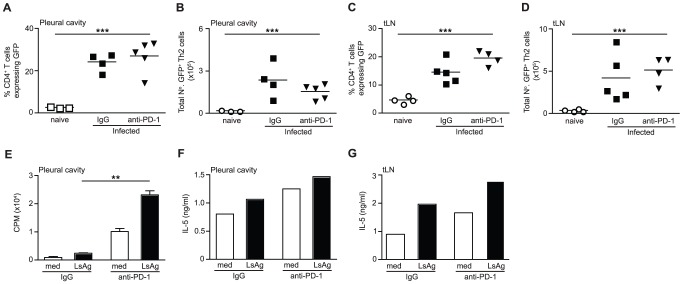
In vivo PD-1 blockade results in a long-term restoration of Th2 cell functional quality. *L. sigmodontis*-infected IL-4gfp reporter mice were treated with a blocking anti-PD-1 mAb (closed triangles) or rat IgG (closed squares) from d 28–d43 and Th2 cell quantity and functional quality assessed at d 60. Open symbols represent naïve untreated controls. (A–D) The proportion (A & C) and total number of IL-4gfp^+^ Th2 cells (B & D) in the PC (A & B) and tLN (C & D). Panels show one out of two representative experiments with 4–6 mice per group. Symbols denote individual mice and lines represent means. *** Significant effect of infection independent of treatment (p<0.001, ANOVA performed on combined data from two independent experiments). (E–G) IL-4gfp^+^ Th2 cells were purified from the PC (E & F) and tLN (G) and their ability to proliferate (E) and produce IL-5 (F & G) following in vitro restimulation with LsAg was assessed. Panels show one representative experiment of two. Within each experiment IL-4gfp^+^ Th2 cells were pooled from 6–10 mice per group. Bars show means (E–G). Due to the pooled samples error bars show SD of triplicate cultures for proliferation (E), and there are no error bars or statistics for IL-5 (F & G). ** Significant effect of treatment upon restimulation with LsAg (p<0.01, ANOVA performed using combined data from two independent experiments).

PD-1 blockade could enhance protective immunity to *L. sigmodontis* by restoring the functional quality of hypo-responsive Th2 cells and/or by increasing the overall quantity of Th2 cells. To address this we treated *L. sigmodontis* infected BALB/c IL-4gfp reporter mice with an anti-PD-1 blocking mAb from d 28–43 pi and quantified the number and antigen-responsiveness of IL-4gfp^+^ Th2 cells at d 60 pi (Figure 4A). No differences were found in the proportion or total number of IL-4gfp^+^ Th2 cells within the PC or tLN at d 60 pi (Figure 5A–D), indicating that PD-1 blockade does not result in a long-term elevation in Th2 cell numbers.

To examine if PD-1 blockade increased the antigen-specific functional quality of the hypo-responsive Th2 cells, IL-4gfp^+^ Th2 cells were purified from the PC and tLN of control and PD-1 treated mice 60 d pi and equal numbers of Th2 cells were restimulated in vitro with LsAg using naïve irradiated splenocytes as APC. Due to the low number of IL-4gfp^+^ T cells present within the PC it was necessary to perform the assays on pooled cells from 6–10 mice. PC IL-4gfp^+^ Th2 cells purified from anti-PD-1 treated mice showed significantly increased antigen-specific proliferation and elevated IL-5 production compared to IL-4gfp^+^ Th2 cells from control mice (Figure 5E and F). PD-1 blockade also increased the capacity of purified tLN IL-4gfp^+^ Th2 cells to secrete IL-5 in response to LsAg at d 60 (Figure 5G). LsAg-specific production of IL-4, IL-10 and IFN-γ by tLN and PC IL-4gfp^+^ Th2 cells was not consistently detectable (data not shown). Thus, PD-1 blockade results in a long-term enhancement of Th2 immunity by augmenting the functional quality of parasite-specific Th2 cells, rather than increasing the overall number of Th2 cells.

### Reprogramming of Th2 cell hypo-responsiveness is preceded by a temporary expansion of IL-4gfp^+^ Th2 cells within the draining LN

**Figure 6:**
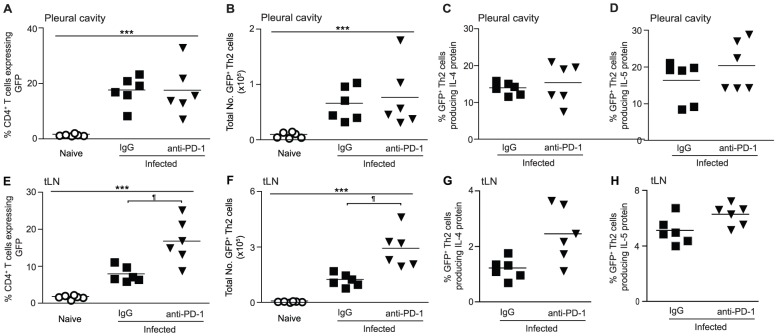
In vivo PD-1 blockade increases the expansion of IL-4gfp^+^ T cells within the tLN immediately post-treatment. *L. sigmodontis*-infected BALB/c IL-4gfp reporter mice were treated with a blocking anti-PD-1 mAb (closed triangles) or rat IgG (closed squares) from d 28–37 and IL-4gfp^+^ T cells from the PC (A–D) and tLN (E–H) analysed at d 40. The proportion (A & E) and total number (B & F) of CD4^+^ T cells expressing GFP, as well as the percentage of IL-4gfp^+^ Th2 cells producing IL-4 (C & G) and IL-5 (D & H) protein following simulation with PMA and ionomycin was assessed. Panels show one representative experiment of three with 4–6 mice per group. Symbols denote individual mice and lines represent means. *** Significant effect of infection independent of treatment (p<0.001, ANOVA performed on combined data from three independent experiments). ¶ Significant pair-wise comparison (p<0.05, Tukey's HSD).

During chronic viral infections PD-1 blockade acts directly on exhausted CD8^+^ T cells to restore their function [28]. Similarly, our data showed that in vitro PD-1 blockade directly enhanced the functional quality of *L. sigmodontis*-specific hypo-responsive IL-4gfp^+^ Th2 cells (Figure 3F and G), and in vivo blockade led to increased antigen-specific responsiveness of IL-4gfp^+^ Th2 cells 20 d after treatment had finished. This predicts that in vivo PD-1 blockade during *L. sigmodontis* infection would initially enhance the functional quality of existing IL-4gfp^+^ Th2 effector cells within the PC. However, when PC Th2 cell responses were assayed immediately following treatment (d 40 pi, Figure 4A) there were no increases in the proportion or total numbers of IL-4gfp^+^ Th2 cells within the PC (Figure 6A and B). There was also no increase in the proportion of IL-4gfp^+^ Th2 cells capable of producing IL-4 or IL-5 protein following stimulation with PMA and ionomycin (Figure 6C and D).

In contrast, PD-1 blockade caused a 2-fold increase in the proportion and total numbers of IL-4gfp^+^ T cells within the tLN (Figure 6E and F), although again had no impact on the proportion of IL-4gfp^+^ Th2 cells producing IL-4 or IL-5 protein (Figure 6G and H). Consistent with the expression pattern of PD-1, the increase in tLN IL-4gfp^+^ T cells was caused by an expansion of both IL-4gfp^+^CXCR5^−^ Th2 cells and IL-4gfp^high^CXCR5^+^ Tfh cells, with IL-4gfp^+^CXCR5^−^ Th2 cells accounting for 44% of the expansion (data not shown). These data suggest that, although in the long-term PD-1 blockade increased the antigen-specific functional quality of IL-4gfp^+^ Th2 cells at the infection site, anti-PD-1 treatment did not recover the responsiveness of IL-4gfp^+^ Th2 cells to PMA and ionomycin. Instead it initially enhanced Type 2 responses within the tLN resulting in a temporary increase in the number of IL-4gfp^+^ Th2 and Tfh cells.

### PD-L2, but not PD-L1, inhibits protective immunity to *L. sigmodontis*

**Figure 7:**
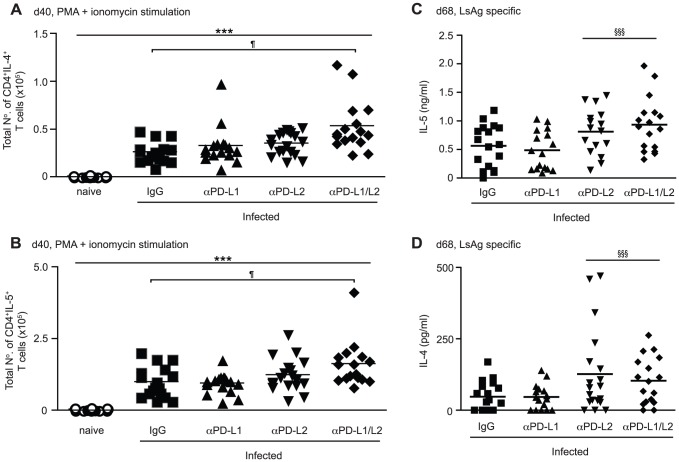
PD-L1 and PD-L2 synergise to regulate Th2 cell immunity during *L. sigmodontis* infection. *L. sigmodontis*-infected BALB/c mice were treated with blocking anti-PD-L1 and anti-PD-L2 mAb alone (up-facing and down-facing triangles respectively) or in combination (diamonds), or with rat IgG (squares) from d 28–37/43 and Th2 responses assessed at d 40 (A & B) and d 68 (C & D). Open symbols represent naïve untreated controls. Symbols denote individual mice and lines represent means. (A & B) Total number of tLN CD4^+^ T cells expressing IL-4 (A) and IL-5 (B) protein at d 40 pi following *ex vivo* stimulation with PMA and ionomycin. Panels show pooled data from three independent experiments. *** Significant effect of infection and treatment (p<0.001, ANOVA performed on combined data from three independent experiments). ¶ Significant pair-wise comparison (p<0.05, Tukey's HSD). (C & D) Production of IL-5 (C) and IL-4 (D) by tLN cells following in vitro stimulation with LsAg. Panels show pooled data from three independent experiments. §§§ Significant treatment effect in the presence of anti-PD-L2 Ab (p<0.001, ANOVA performed on combined data from three independent experiments).

PD-1 interacts with two ligands, PD-L1 and PD-L2 [44], and to identify through which ligand PD-1 was acting *L. sigmodontis* infected BALB/c mice were treated with blocking anti-PD-L1 and/or anti-PD-L2 mAbs [45] from d 28–43 pi (Figure 4A). At d 40 pi, when PD-1 blockade results in an expansion of IL-4gfp^+^ Th2 cells within the tLN (Figure 6E and F), significantly increased numbers of IL-4 and IL-5 producing CD4^+^ T cells were found following combined blockade of PD-L1 and PD-L2 (Figure 7A and B). Blockade of PD-L1 or PD-L2 alone had no effect on the numbers of IL-4 and IL-5 producing CD4^+^ T cells, indicating that PD-L1 and PD-L2 synergise to regulate the expansion of IL-4 and IL-5 secreting T cells within the tLN.

In contrast, blockade of PD-L2 alone was sufficient to enhance resistance and impair the development of patent infections, demonstrated by a significant two-fold reduction in the incidence of mice with Mf within their blood at d 68 pi compared to IgG controls (Table 2). This was associated with a significantly reduced incidence of Mf in the pleural cavity, suggesting a lower release of Mf by female parasites. Interestingly, although mice treated with both anti-PD-L1 and anti-PD-L2 had a lower incidence of blood Mf, PD-L1 neutralisation alone resulted in a trend towards an increased number of mice harbouring blood Mf. This suggests that PD-L1 may promote rather than inhibit protective immunity to *L. sigmodontis*, but that the inhibitory role of PD-L2 is dominant. Whilst anti-PD-L2 treatment significantly reduced the number of mice developing patent infections, those that presented with circulating Mf had similar levels within the blood and pleural cavity to the IgG control group (Figure S2B and C). The increased resistance resulting from blockade of PD-L2 was associated with significantly elevated production of IL-5 protein (Figure 7C), as well as IL-4 (Figure 7D), by tLN cells restimulated in vitro with LsAg. Thus, while PD-L1 and PD-L2 act synergistically to control Th2 cell expansion in the tLN, PD-L2 plays the dominant role in dampening IL-4 and IL-5 production and resistance towards *L. sigmodontis*.

**Table 2:** In vivo blockade of PD-L2, but not PD-L1, results in a reduced incidence of Mf within the blood and pleural cavity.

	Incidence of blood Mf				Incidence of PC Mf			
	IgG	αPD-L1	αPD-L2	αPD-L1 ^+^ αPD-L2	IgG	αPD-L1	αPD-L2	αPD-L1 ^+^ αPD-L2
Exp 1	3/6	3/3	1/6	2/5	4/6	3/3	1/6	1/5
Exp 2	3/6	5/6	1/6	2/5	3/6	4/5	4/6	3/5
Exp 3	5/8	5/7	4/7	3/7	7/8	7/7	4/7	3/7
Total	11/20	13/16	6/19*	7/17*	14/20	14/15	9/19*	7/17*
	(55%)	(81%)	(32%)	(41%)	(70%)	(93%)	(47%)	(41%)

* Significant difference in Mf incidence driven by αPD-L2 treatment, p<0.001 (GLM using combined data from three experiments).

### *L. sigmodontis*-elicited alternatively activated macrophages do not inhibit Th2 cells via the PD-1 pathway

**Figure 8:**
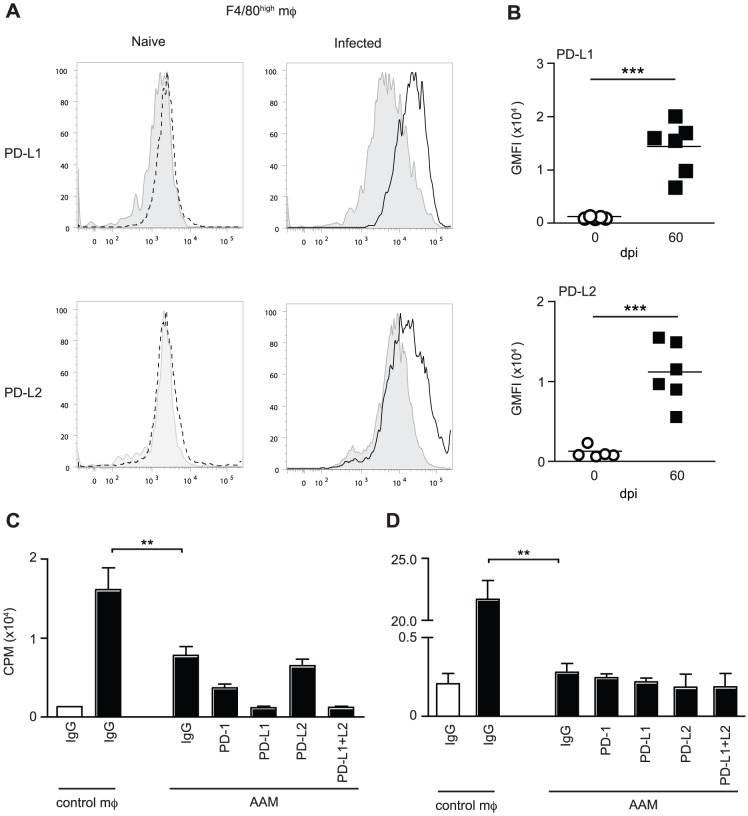
*L. sigmodontis*-elicited alternatively activated macrophages do not suppress T cells via the PD-1 pathway. PC macrophages from *L. sigmodontis* infected or naïve BALB/c mice were assessed for expression of PD-L1 and PD-L2 and suppressive function at d 60 pi. (A) Representative histograms showing expression of PD-L1 and PD-L2 on PC F4/80^high^ macrophages. Grey shaded histograms represent isotype controls, dotted lines show naïve macrophages and solid lines show infected macrophages. (B) Geometric mean fluorescent intensity minus isotype controls of PD-L1 and PD-L2 on F4/80^high^ macrophages from naïve (open circles) and infected (closed squares) mice. Symbols represent individual animals and lines show mean. Panels show one representative experiment of two with at least 5 mice per group. *** Significant effect of infection, (p<0.001, ANOVA based on combined data from two independent experiments). (C & D) Adherent PC macrophages were purified from individual naïve and *L. sigmodontis* infected mice and tested in vitro for their ability to inhibit the Ag-specific proliferation of pooled PC IL-4gfp^+^ Th2 cells purified 60 d pi (C) or naïve DO11.10 T cells (D) in the presence of control IgG or blocking antibodies against PD-1, PD-L2, and PD-L2. Panels show mean and SD with 6 mice per group. Open bars show medium control and closed bars represent the presence of LsAg (C) or OVA (D). ** p<0.001 (ANOVA using combined data from two independent experiments).

Alternatively activated macrophages can inhibit Th2 responses to helminths through PD-L1 and PD-L2 [37] or PD-L2 alone [35]. Expression of PD-L1 on macrophages, independent of alternate activation, also inhibits T cell responses to *S. mansoni* [36]. AAM are elicited during *L. sigmodontis* infection resulting in T cell suppression [16], suggesting that they could drive the hypo-responsive Th2 cell phenotype via PD-L1 or PD-L2. To test this, the expression of PD-L1 and PD-L2 on F4/80^high^ pleural cavity AAM was assessed during *L. sigmodontis* infection. Analysis was performed at d 60 pi when PC derived F4/80^high^ macrophages are known to be alternatively activated [16]. Although F4/80^high^ macrophages from naïve mice did not express PD-L1 or PD-L2 constitutively, levels of both were up-regulated 11-fold following infection (Figure 8A and B). To determine whether AAM inhibit Th2 cells via PD-L1 and/or PD-L2, we purified and restimulated d 60 PC IL-4gfp^+^CD4^+^ Th2 cells with LsAg in the presence of d 60 PC AAM or naïve control macrophages. Irradiated naïve splenocytes were used as APC. The ability of the hypo-responsive IL-4gfp^+^ Th2 cells to proliferate in response to LsAg was then assessed following the addition of neutralising antibodies to PD-1, PD-L1 and PD-L2. In the presence of AAM, the LsAg-specific proliferation of IL-4gfp^+^CD4^+^ Th2 cells was significantly reduced compared to culture with naïve control macrophages confirming that the AAM were suppressive (Figure 8C). However, blocking PD-1, PD-L1, PD-L2, or a combination of PD-L1 and PD-L2, failed to restore LsAg-specific proliferation (Fig. 8C), indicating that AAM-mediated suppression of proliferation was not via the PD-1 pathway. Similarly, *L. sigmodontis*-elicited AAM inhibited the OVA-specific proliferation of naïve DO11.10 T cells independently of PD-1, PD-L1 and PD-L2 (Figure 8D). Consistent with our previous work showing that AAM only inhibit T cell proliferation and not cytokine production [16], the addition of AAM did not reduce Th2 cell production of IL-5 or IL-4 (data not shown). Thus, *L. sigmodontis*-elicited AAM do not suppress the antigen-specific proliferation of committed Th2 cells or naïve T cells via the PD-1 pathway.

## Discussion

Suppression of protective immunity during helminth infections is known to involve a wide range of Th2 cell extrinsic immune regulators [2], [12]. However, the intrinsic fate of parasite-specific Th2 cells within a chronic immune down-regulatory environment, and the resultant impact such fate changes may have on host resistance is unknown. In this study we used IL-4gfp reporter mice to demonstrate that during chronic filarial nematode infection CD4^+^ Th2 cells are conditioned towards an intrinsically hypo-responsive phenotype, characterised by a loss of functional ability to proliferate and produce IL-4, IL-5 and IL-2 cytokines. The development of Th2 cell hypo-responsiveness was a key element in determining susceptibility to *L. sigmodontis* infection, and could be reversed in vivo by blockade of the PD-1/PD-L2 pathway resulting in the long-term recovery of Th2 cell functional quality and enhanced resistance.

The hypo-responsive Th2 cell phenotype during *L. sigmodontis* infection had some parallels with T cell exhaustion, which leads to impaired Th1 immunity towards viruses [28] and protozoan parasites [29], [30]. Similar to exhaustion, Th2 cell hypo-responsiveness was mediated through PD-1 co-inhibition, and was characterised by a sequential loss of cytokine production with IL-4 being lost prior to IL-5. In the future it will be important to confirm whether Th2 cell hypo-responsiveness also extends to other Th2 cytokines, such as IL-13, as limited cell numbers restricted the cytokines we could measure in this study. Alongside the similarities to exhaustion there were also notable differences. PD-1 predominantly mediates CD8^+^ T cell exhaustion via interactions with PD-L1 [28], [30], whereas Th2 hypo-responsiveness was driven through interactions with PD-L2. This preferential regulation of Type 2 immunity by PD-L2 is consistent with its expression being specifically induced by IL-4 and STAT-6 signalling, contrasting with PD-L1, which is preferentially regulated by Type 1 stimuli [46], [47]. Also, PD-1 interactions with PD-L1 are actively required for maintaining exhaustion, with PD-L1 blockade immediately boosting the functional quality of exhausted CD8^+^ T cells [28]. Although in vitro PD-1 blockade enhanced the antigen-specific ability of hypo-responsive Th2 cells to produce Th2 cytokines, stimulating them with PMA and ionomycin, which bypasses the PD-1 pathway, failed to recover their function. Similarly, the Th2 cells remained hypo-responsive to PMA and ionomycin stimulation immediately following in vivo PD-1 blockade, although it led to a recovery in their functional ability to respond to parasite antigens later in infection. Thus, the hypo-responsive Th2 cell phenotype is likely distinct from exhaustion, and appears to be more deep-seated, involving more mechanisms, than PD-1 co-inhibition alone.

The disparity between GFP expression, which marks IL-4 mRNA, and IL-4 protein suggests that the loss of cytokine production by hypo-responsive Th2 cells is due to post-transcriptional regulation, which is an essential step in the production of Th2 cytokines [32]. This has parallels with anergic self-reactive T cells that express mRNA for effector cytokines such as IFN-γ, IL-4, and IL-13, but are unable to produce protein because translation is blocked by AU-rich elements within the cytokine 3′UTRs [48]. The description of an anergic molecular signature within the PBMC of filariasis patients and the findings that addition of IL-2 can restore the in vitro immune responsiveness of human PBMC [3], [49], reinforce the idea that Th2 hypo-responsiveness is a form of anergy. If so, it is more likely to represent a form of adaptive tolerance than classical clonal anergy as it is not rescued by stimulation with PMA and ionomycin, and results in the shutdown of multiple cytokines, not just IL-2 [50]. Similarly, during *S. mansoni* infection the anergy factor GRAIL is responsible for driving Th2 cells towards an intrinsically hypo-responsive state with characteristics of adaptive tolerance [24]. Interestingly, there are differences in Th2 cell hypo-responsiveness during filariasis and schistomiasis. Firstly, GRAIL is not part of the anergic signature of PBMC from filariasis patients [3]. Secondly, *S. mansoni* induced Th2 cell hypo-responsiveness does not relate to PD-1 [24]. Thus, while an intrinsic functional shut-down of Th2 cells appears common to different chronic helminth infections, it may involve distinct mechanisms.

Consistent with the hypothesis that multiple factors maintain Th2 cell hypo-responsiveness, in vivo PD-1 blockade failed to initially expand or recover the function of the hypo-responsive Th2 cells at the infection site. Instead it first caused a temporary expansion of CXCR5^−^IL-4gfp^+^ Th2 cells and CXCR5^+^ IL-4gfp^+^ Tfh cells within the draining LN, followed by the appearance of functionally superior IL-4gfp^+^ Th2 cells at the infection site 20 days later. In contrast to the PC, CD4^+^ T cells in the LN did not lose the ability to produce IL-4 protein during *L. sigmodontis* infection. Tfh cells are the predominant source of IL-4 in the LN and demonstrate distinct control of IL-4 gene expression compared to Th2 cells [39], [40], [41], [51]. Thus, similar to viral infections where Tfh cells do not become exhausted [52], Tfh cells may remain functionally responsive during chronic helminth infection and maintain a source of IL-4. It is interesting to speculate that, rather than directly rescuing the hypo-responsive Th2 cells, PD-1 blockade acted by expanding a reservoir of still responsive IL-4gfp^+^ T cells within the LN, either Tfh or Th2 cells, that over time replaced the unresponsive Th2 cells at the infection site. Alternatively, PD-1 can inhibit T cell priming [53] and so its blockade may have favoured the generation of new responsive Th2 cells.

The involvement of PD-L2, rather than PD-L1, indicates that professional immune cells regulate CD4^+^ Th2 cell hypo-responsiveness. Suppression of T cell responses by PD-1 during helminth infections has mainly been attributed to macrophages expressing PD-L1 and/or PD-L2, and the PD-1 pathway has been shown to be an important mechanism of suppression by AAM [35], [36]. Although *L. sigmodontis* infection induces suppressive AAM [16], the proliferative suppression of Th2 cells and naïve T cells by *L. sigmodontis*-elicited AAM was independent of PD-1, PD-L1 and PD-L2. Thus, whilst AAM are clearly able to suppress T cells via the PD-1 pathway they do not do so in all Th2 contexts, and it is not their dominant mechanism of suppression during *L. sigmodontis* infection. Furthermore, as we have previously shown [16], suppression by *L. sigmodontis*-elicited AAM was restricted to T cell proliferation and did not inhibit the production of Th2 cell cytokines. As reduced Th2 cytokines were a defining characteristic of hypo-responsive Th2 cells it indicates that AAM are not driving hypo-responsiveness, although in vivo AAM studies are required to confirm our in vitro findings. Interestingly, B cell deficient mice are more resistant to primary *L. sigmodontis* infection indicating a regulatory role for B cells [54]. B cells can express PD-L2 [44] and up-regulate it during *L. sigmodontis* infection (van der Werf, Taylor, unpublished data) raising the possibility that B cells are involved in conditioning Th2 cells towards hypo-responsiveness. Alternate candidates that may influence the intrinsic functional quality of Th2 cells include DC and Foxp3^+^ Tregs.

The development of functionally impaired CD4^+^ Th2 cells provides a potential explanation for why protective memory to helminths takes decades to develop in humans [14]. Hypo-responsive Th2 cells may fail to develop into memory cells as seen with exhausted CD8^+^ T cells [22], and consistent with this filariasis patients show contractions in their central memory CD4^+^ T cell pool [55]. Alternatively, a tolerised memory response may develop as anergic T cells can show long-term survival and maintain their unresponsive phenotype even in the absence of antigen [56]. A failed or tolerised memory response may also explain why helminth-infected individuals become rapidly re-infected following drug clearance, even though some aspects of immune suppression are lifted. PD-1 blockade in combination with drug treatments may thus represent a new strategy for restoring protective Th2 memory, particularly as we find PD-1 blockade has a long-term effect on Th2 cell quality and it has been successfully used in clinical trials to treat cancer [57], [58]. Alternate targets include GITR, as providing co-stimulation through GITR increases the functional quality of *L. sigmodontis* specific Th2 cells [59], and CTLA-4, which promotes the expression of T cell anergy factors and inhibits protective Th2 immunity during filarial infections [3], [26]. The development of Th2 hypo-responsiveness also has implications for vaccine development. Even the best live-attenuated filarial vaccines are only 70% effective [60], meaning that residual infections could condition vaccine-elicited Th2 cells towards hypo-responsiveness resulting in vaccine failure.

Altogether, our data demonstrates that intrinsic changes in Th2 cell quality lead to the development of a functionally hypo-responsive phenotype that plays a key role in determining susceptibility to filarial nematode infection, and that can be therapeutically manipulated to promote resistance. Alongside its relevance to the treatment of helminth infections, a deeper understanding of how Th2 cells are conditioned towards hypo-responsiveness will help define the checkpoints that determine whether a T cell remains inflammatory or becomes tolerised during chronic immune challenge. This may help determine why Th2 cells fail to shutdown naturally in settings of chronic pathology, such as in allergic inflammation or fibrosis, and potentially lead to novel approaches for tolerising pathogenic Th2 cells.

## Materials and Methods

### Ethics statement

All animal work was approved by the University of Edinburgh Ethics Committee (PL02-10) and by the UK Home Office (PPL60/4104), and conducted in accordance with the Animals (Scientific Procedures) Act 1986.

### Animals and parasites

Female BALB/c and IL-4gfp 4get reporter mice on the BALB/c background (courtesy of Markus Mohrs, The Trudeau Institute) [27] were bred in-house and maintained under specific pathogen-free conditions at the University of Edinburgh. Mice were used at 6–12 weeks of age. The *L. sigmodontis* life cycle was maintained in gerbils using the mite vector *Ornithonyssus bacoti* [61]. Mice were infected s.c. on the upper back with 30 *L. sigmodontis* L3 larvae. Adult parasites were recovered by lavage and fixed in 70% ethanol for morphological analysis. The analysis of fecundity of female *L. sigmodontis* parasites was performed as previously [43]. The numbers of healthy eggs and Mf within the anterior, median and posterior of the uterus were semi-quantitatively scored on scales of 0–5. Each region's scores were summed giving a total possible score of 15. To quantify blood Mf, 30 µL of tail blood was collected in FACS lysing solution (Becton-Dickinson). *L. sigmodontis* antigen (LsAg) was prepared by collecting the PBS-soluble fraction of homogenized adult male and female worms.

### *In vivo* antibody treatments

Mice received i.p. injections of 250 µg of blocking anti-PD-1 mAb (RMP1-14, Bioxcell), 250 µg of blocking anti-PD-L2 mAb (Ty25, Bioxcell) or 200 µg of blocking anti-PD-L1 mAb (MIH5, in house) every three days from d28–43 pi An equivalent dose of rat IgG (Sigma-Aldrich) was used as control.

### Cell purifications and *in vitro* restimulations

The parathymic, posterior, mediastinal and paravertebral LN, were taken as a source of tLN draining the PC. PC cells were recovered by lavage. TLN and spleen cells were dissociated and washed in RPMI-1640 (invitrogen) supplemented with 0.5% mouse sera (Caltag-Medsystems), 100 U/ml penicillin, 100 µg/ml streptomycin and 2 mM L-glutamine. To purify GFP^+^CD4^+^ T cells from IL-4gfp mice PC or tLN cells were enriched for CD4^+^ T cells by magnetic negative selection (DynaMag, Dynal) using anti-CD8 (53–6.72), anti-B220 (RAB632), anti-MHC class II (M5/114.15.2), anti-Gr1 (RB6-8C5) and anti-F4/80 (A3-1), followed by sheep anti-rat IgG Dynal Beads (Invitrogen). Cells were stained with allophycocyanine-conjugated anti-CD4 (RM4-5). To purify GFP^+^CD4^+^ T cells from anti-PD-1 treated mice cells were stained with phycoerythrin-conjugated anti-CD4 followed by positive magnetic section with anti-phycoerythrin MicroBeads (Milenyi Biotec). GFP^+^CD4^+^ T cells were then purified using a FACSAria flow sorter (Becton-Dickinson). On average, sorted cells were 98.3% positive for CD4, of which 97.6% were GFP^+^. Due to limited cell numbers it was necessary to pool CD4^+^GFP^+^ T cells from 10–15 mice to obtain sufficient numbers. Whole tLN cells were cultured at 5×10^5^ cells/well and spleen cells at 1×10^6^ cells/well in 96 well plates (Nunc). Purified GFP^+^CD4^+^ T cells were cultured at 5–10×10^4^ cells/well with 1×10^6^ irradiated (30 Gy) naïve splenocytes. For in vitro restimulations, cells were cultured in medium alone or with 10 µg/ml LsAg for 72 hours followed by addition of 1 µCi/well [Methyl-^3^H]-Thymidine (PerkinElmer) for 16 h to measure proliferation. Blocking antibodies against PD-1, PD-L1 and PD-L2 were used at 20 µg/ml as detailed in the results. For macrophage suppression assays, PC cells were adhered to 96-well flat-bottom plates at 1×10^5^ cells/well for 2 h at 37°C and the non-adherent fraction rinsed off. GFP^+^CD4^+^ T cells or DO11.10 CD4^+^ T cells were added at 5×10^4^ cells/well and after 72 h the cultures were pulsed with thymidine as described. DO11.10 cells were restimulated with 0.5 µg/ml OVA peptide (ISQAVHAAHAEINEAGR) from Advanced Biotechnology Centre (Imperial School of Medicine, London, U.K.). For measurement of intra-cellular cytokines cells were stimulated for 4 hours with 0.5 µg/ml PMA (Sigma-Aldrich) and 1 µg/ml Ionomycin, with 10 µg/ml Brefeldin A added for the final 2 hours (all from Sigma-Aldrich).

### Flow cytometry and ELISA

The following antibodies were used: Alexafluor700-conjugated anti-CD4 (RM4-5), polyclonal anti-GFP (Ebioscience), Alexafluor488-conjugated goat anti-rabbit IgG (Invitrogen), eFluor450-conjugated anti-IL-2 (JES6-5H4, Ebioscience), phycoerythrin-conjugated anti-IL-4 (11B11, Biolegend), allophycocyanine-conjugated anti-IL-5 (TRFK5, Biolegend), phycoerythrin-conjugated or biotinylated anti-PD-1 (J43, Ebioscience), biotinylated anti-PD-L1 (MIH5, Ebioscience), phycoerythrin-conjugated anti-PD-L2 (Ty25, Ebioscience), biotinylated anti-CXCR5 (RF8B2, BD Biosciences) and allophycocyanine-conjugated streptavidin (Biolegend). Non-specific binding was blocked with 4 µg of rat IgG/1×10^6^ cells. For intracellular cytokine staining dead cells were excluded using Aqua Dead Cell Stainkit (Molecular Probes), and the cells fixed and permeabilized using the BD Cytofix/Cytoperm kit. Staining was compared with the relevant isotype controls to verify specificity. Flowcytometric acquisition was performed on a FACSCANTO II or LSR II (BD Biosciences) and data were analyzed using Flowjo Software (Tree Star). Antibody pairs used for cytokine ELISA were as follow: IL-4 (11B11/BVD6-24G2) and IL-5 (TRFK5/TRFK4). Recombinant murine IL-4 and IL-5 (Sigma-Aldrich) were used as standards. Biotin detection antibodies were used with ExtrAvidin-alkaline phosphatase conjugate (Sigma-Aldrich) and Sigma Fast *p*-nitrophenyl phosphate substrate (Sigma-Aldrich).

### Statistics

Statistical analysis was performed using JMP version 8 (SAS). Parametric analysis of combined data from multiple repeat experiments, or of experiments containing more than two groups, was performed using ANOVA followed by Tukey's post-hoc tests when required. When using two-way ANOVA to combine data from multiple experiments, experimental effects were controlled for in the analysis and it was verified that there were no significant qualitative interactions between experimental and treatment effects. Mf incidence was analysed using a GLM with a binomial distribution.

## Supporting Information

Figure S1 **CD4^+^ Th2 cells lose their functional ability to produce IL-5 and IL-2 during** ***L. sigmodontis*** **infection.** PC CD4^+^ T cells from naive and *L. sigmodontis* infected BALB/c IL-4gfp mice were analysed at d20, d40, and d60 pi for expression of GFP, IL-5 and IL-2. Representative flow plots showing expression of IL-5 (A) and IL-2 (B) by PC IL-4gfp^+^ Th2 cells.
(PDF)

Figure S2 ***L. sigmodontis*** **infected BALB/c IL-4gfp reporter mice were treated with blocking anti-PD-1, anti-PD-L1, anti-PD-L2, or rat IgG from d28 to d43 pi.** (A) Number of Mf within the uteri of individual female parasites recovered from anti-PD-1 (down triangles) and IgG (squares) treated hosts 60 d pi. (B–C) Mf counts per ml of blood (B) and total number of MF within the PC (C) 68 d post-*L. sigmodontis* infection following treatment with IgG (squares), anti-PD-L1 (up triangles), anti-PD-L2 (down triangles) or anti-PD-L1 and anti-PD-L2 in combination (diamonds).
(PDF)
