# Supplementary material for: Th2 Cell-Intrinsic Hypo-Responsiveness Determines Susceptibility to Helminth Infection
Source: PLoS Pathog. 2013 Mar 14;9(3):e1003215. doi: 10.1371/journal.ppat.1003215 (PMC3597521; doi:10.1371/journal.ppat.1003215)
Supplement: Figure S1 — CD4+ Th2 cells lose their functional ability to produce IL-5 and IL-2 during L. sigmodontis infection. PC CD4+ T cells from naive and L. sigmodontis infected BALB/c IL-4gfp mice were analysed at d20, d40, and d60 pi for expression of GFP, IL-5 and IL-2. Representative flow plots showing expression of IL-5 (A) and IL-2 (B) by PC IL-4gfp+ Th2 cells. (PDF) [file ppat.1003215.s001.pdf]

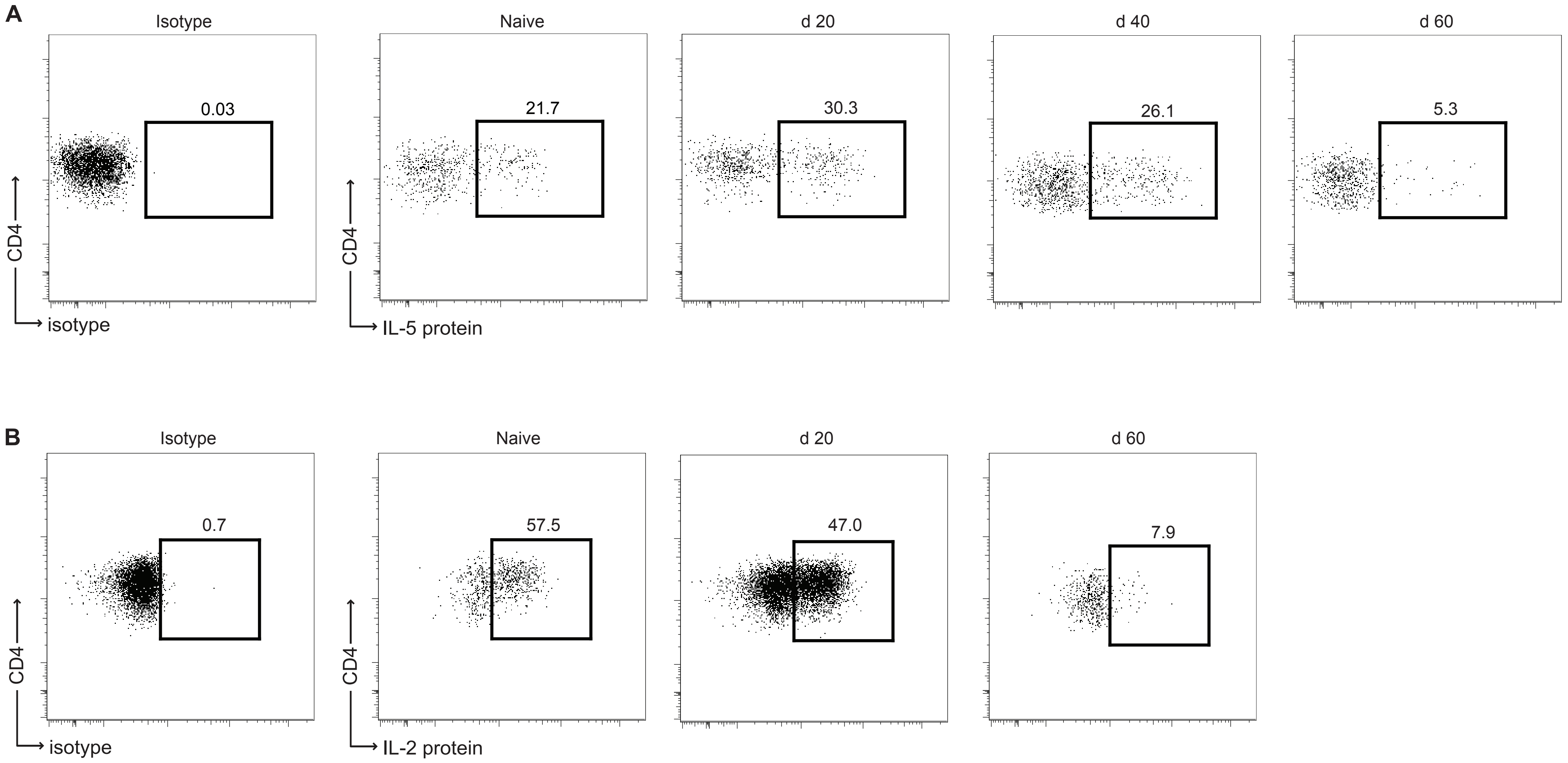

Figure S1. CD4+ Th2 cells loose their functional ability to produce IL-5 and IL-2 during *L. sigmodontis* infection. PC CD4+ T cells from naive and *L. sigmodontis* infected BALB/c IL-4gfp mice were analysed at d20, d40, and d60 pi for expression of GFP, IL-5 and IL-2. Representative flow plots showing expression of IL-5 (A) and IL-2 (B) by PC IL-4gfp+ Th2 cells.
