# Supplementary material for: Th2 Cell-Intrinsic Hypo-Responsiveness Determines Susceptibility to Helminth Infection
Source: PLoS Pathog. 2013 Mar 14;9(3):e1003215. doi: 10.1371/journal.ppat.1003215 (PMC3597521; doi:10.1371/journal.ppat.1003215)
Supplement: Figure S2 — L. sigmodontis infected BALB/c IL-4gfp reporter mice were treated with blocking anti-PD-1, anti-PD-L1, anti-PD-L2, or rat IgG from d28 to d43 pi. (A) Number of Mf within the uteri of individual female parasites recovered from anti-PD-1 (down triangles) and IgG (squares) treated hosts 60 d pi. (B–C) Mf counts per ml of blood (B) and total number of MF within the PC (C) 68 d post-L. sigmodontis infection following treatment with IgG (squares), anti-PD-L1 (up triangles), anti-PD-L2 (down triangles) or anti-PD-L1 and anti-PD-L2 in combination (diamonds). (PDF) [file ppat.1003215.s002.pdf]

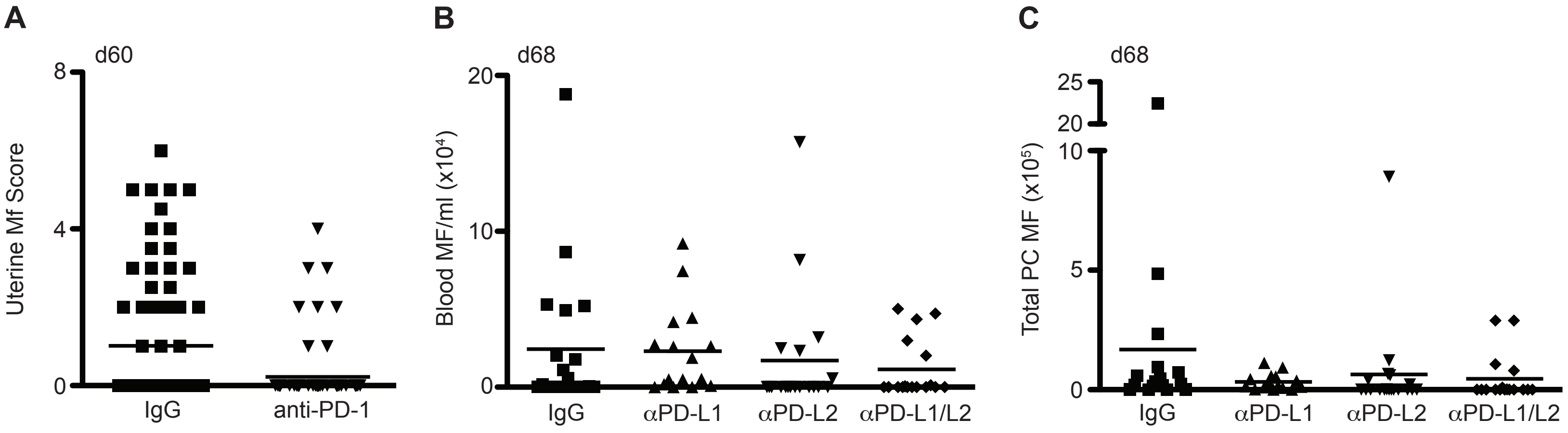

Figure S2. *L. sigmodontis* infected BALB/c IL-4gfp reporter mice were treated with blocking anti-PD-1, anti-PD-L1, anti-PD-L2, or rat IgG from d28 to d43 pi. (A) Number of Mf within the uteri of individual female parasites recovered from anti-PD-1 (down triangles) and IgG (squares) treated hosts 60 d pi. (B - C) Mf counts per ml (B) and total number of MF within the PC (C) 68 d post-*L. sigmodontis* infection following treatment with IgG (squares), anti-PD-L1 (up triangles), anti-PD-L2 (down triangles) or anti-PD-L1 and anti-PD-L2 in combination (diamonds).
